# Associations Between Fine Particulate Matter Components and Daily Mortality in Nagoya, Japan

**DOI:** 10.2188/jea.JE20150039

**Published:** 2016-05-05

**Authors:** Kayo Ueda, Makiko Yamagami, Fumikazu Ikemori, Kunihiro Hisatsune, Hiroshi Nitta

**Affiliations:** 1 Department of Engineering, Graduate School of Kyoto University, Kyoto, Japan; 1 京都大学大学院工学研究科; 2 Nagoya City Institute for Environmental Sciences, Nagoya, Japan; 2 名古屋市環境科学調査センター; 3 Environmental Epidemiology Section, Center for Environmental Health Sciences, National Institute for Environmental Studies, Tsukuba, Japan; 3 国立環境研究所

**Keywords:** particulate matter, chemical components, mortality, air pollution, 粒子状物質, 化学成分, 死亡, 大気汚染

## Abstract

**Background:**

Seasonal variation and regional heterogeneity have been observed in the estimated effect of fine particulate matter (PM_2.5_) mass on mortality. Differences in the chemical compositions of PM_2.5_ may cause this variation. We investigated the association of the daily concentration of PM_2.5_ components with mortality in Nagoya, Japan.

**Methods:**

We combined daily mortality counts for all residents aged 65 years and older with concentration data for PM_2.5_ mass and components in Nagoya from April 2003 to December 2007. A time-stratified case-crossover design was used to examine the association of daily mortality with PM_2.5_ mass and each component (chloride, nitrate, sulfate, sodium, potassium, calcium, magnesium, ammonium, elemental carbon [EC], and organic carbon [OC]).

**Results:**

We found a stronger association between mortality and PM_2.5_ mass in transitional seasons. In analysis for each PM_2.5_ component, sulfate, nitrate, chloride, ammonium, potassium, EC, and OC were significantly associated with mortality in a single-pollutant model. In a multi-pollutant model, an interquartile range increase in the concentration of sulfate was marginally associated with an increase in all-cause mortality of 2.1% (95% confidence interval, −0.1 to 4.4).

**Conclusions:**

These findings suggest that some specific PM components have a more hazardous effect than others and contribute to seasonal variation in the health effects of PM_2.5_.

## Abstract (ja)

**背景：:**

微小粒子状物質(PM2.5)の死亡に対する影響推定値には、季節変動や地域差がみられ、この変動にはPM2.5の化学成分の違いが関係している可能性がある。本研究では、名古屋市においてPM2.5の成分濃度と死亡との関連について検討した。

**方法：:**

名古屋市の2003年4月から2007年12月までにおける65歳以上の日死亡数とPM2.5重量濃度及び成分濃度のデータを結合した。時間層別化ケースクロスオーバーデザインを用い、日々の死亡とPM2.5重量濃度及びそれぞれの成分（塩化物イオン、硝酸イオン、硫酸イオン、ナトリウムイオン、カリウムイオン、カルシウムイオン、マグネシウムイオン、アンモニウムイオン、元素状炭素、有機炭素）との関連について検討した。

**結果：:**

PM2.5重量濃度と死亡との関連は、春・秋により大きかった。PM2.5成分濃度については、単汚染物質モデルで、硫酸イオン、硝酸イオン、塩化物イオン、アンモニウムイオン、カリウムイオン、元元素状炭素、有機炭素が死亡と有意な関連がみられた。多汚染物質モデルでは、硫酸イオンの四分位範囲濃度上昇あたり、2.1%(95%信頼区間、-0.1, 4.4%)の全死亡増加と関連しており、有意に近い関連であった。

**結論：:**

これらの結果から、ある粒子状物質の成分は他の成分より有害な影響が大きく、粒子状物質の健康影響の四季変動に関わっていることを示唆した。

## INTRODUCTION

Epidemiological studies have shown an association between mortality and short-term exposure to particulate matter (PM). PM is a complex mixture of particles with various sizes and chemical compositions, and there is interest in whether this variation is responsible for differences in target health outcomes and effect size estimates. It has been postulated that fine particles have greater health effects than coarse particles, because fine particles, which are primarily derived from fossil fuel combustion, deposit more deeply in the lungs. Previous studies have shown that fine PM with an aerodynamic diameter less than 2.5 µm (PM_2.5_) has a strong effect on mortality.^1^^,^^2^

Several multi-city studies have suggested regional heterogeneity in the estimated effect of PM on mortality^3^^–^^5^ and hospitalization.^6^^,^^7^ Seasonal variation has also been found to affect estimates of PM effects.^6^^,^^8^ These regional and seasonal variations are partly explained by community characteristics, such as air-conditioning prevalence,^8^ population density,^9^ proportion of elderly residents,^10^ and effect modification by ambient temperature.^11^ Seasonal and regional variations in the chemical composition of ambient particles have also been suggested to contribute to heterogeneity in PM health effects. Using seasonal mean concentrations of particle components, Franklin et al indicated that specific PM_2.5_ components modified the association between mortality and mass concentration of PM.^12^ Bell et al also illustrated the role of PM composition in the effect of PM_2.5_ on hospital admission in the United States.^6^

Several studies have examined the association between particle components and mortality in the United States,^13^^–^^16^ Chile,^17^ Korea,^18^^,^^19^ and China.^20^ However, no such study has been conducted in Japan. In the present study, we investigated the association of daily concentrations of PM components with mortality in Nagoya, a large urban city located on the Pacific coast of Japan.

## METHODS

### Mortality data

Data on daily mortality counts, which were provided by the Ministry of Health, Labour and Welfare of Japan, were collected among all Nagoya residents aged 65 years and older from April 2003 to December 2007. Mortality records included information on sex, age, and date of death. Primary cause of death was coded according to the International Classification of Diseases, 10th revision. Analyses were performed for all-cause deaths except injuries and external reasons (A00–R99), cardiovascular disease (I00–I99), and respiratory disease (J00–J99). This study was approved by the Ethics Committee of the Graduate School of Engineering, Kyoto University.

### Environmental data

Details of the study site and ambient air sampling methods are described by Yamagami et al.^21^ Briefly, daily samples of PM_2.5_ were collected from April 2003 to December 2007 at a monitoring site located at the Nagoya City Institute for Environmental Sciences, which is located in the southern part of Nagoya city, approximately 110 m north of National Route 23. PM_2.5_ concentrations were simultaneously measured at three other monitoring sites within the city for each season during 2009. Variation in PM components was temporally homogeneous between each sampling site within 7 km. Moreover, PM_2.5_ mass concentrations at the Nagoya City Institute were highly correlated with those measured at the monitoring sites 5 and 10 km away from the study site (*r* = 0.94 and 0.90, respectively). Thus, the PM_2.5_ mass concentrations and the components measured at the study site were considered representative of those in the southern area of Nagoya City. Most data were collected from Sunday through Thursday using a pair of FRM-2000 samplers (Rupprecht & Patashnick, Albany, NY, USA) with PTFE filters (TK15-G3M; Pall Life Sciences, Port Washington, NY, USA) for ion components and Quartz fiber filters (2500QAT-UP; Pall Life Sciences) for carbon. Samples were collected from 9:30 a.m. through 9:00 a.m. on the next day. We used the concentration of each PM component sampled from 9:30 a.m. until 9:00 a.m. of the next day as a proxy of concentration at lag 0. In order to verify that the concentration of PM components sampled from 9:30 a.m. until 9:00 a.m. of the next day can be used as a proxy of concentration from 0 to 23 hours of the day, we obtained hourly samples of PM_2.5_ monitored by Tapered Element Oscillating Microbalance in 2003 at the site 6.5 km away from the study site. We calculated 24-hour mean concentration using the hourly values from 0 to 23 of the day and compared the proxy concentration with the 24-hour mean concentration from 9 a.m. until 9 a.m. of the next day. Pearson’s correlation coefficient was 0.92 (eFigure 1).

During the study period (1736 days), the number of days with available data on PM components ranged from 886 to 926 days. Ion chromatography (Dionex ICS-1000; Thermo Fisher Scientific Inc., Waltham, MA, USA) was used for analysis of ion components (chloride, nitrate, sulfate, ammonium, sodium, potassium, calcium, and magnesium). A thermal/optical carbon analyzer (Sunset Laboratory Inc., Tigard, OR, USA) with the IMPROVE thermal/optical reflectance protocol was used for analysis of organic carbon (OC) and elemental carbon (EC). Values below the detection limit were recorded as half of the detection limit.

Hourly concentrations of nitrogen dioxide (NO_2_) and photochemical oxidants (Ox), which are mixtures of ozone and other secondary oxidants generated by photochemical reactions, were collected at the closest monitoring station to the Nagoya City Institute for Environmental Sciences. Data on meteorological variables were obtained from the Japan Meteorological Agency, and hourly measurements were collected at the Nagoya District Meteorological Observatory. Daily mean ambient temperature, relative humidity, and concentration of NO_2_ were calculated using hourly measurements from 0 to 23 hours. Daily maximum 8-hour mean concentration of Ox was also calculated. Data were excluded from days when more than four measurements were missing.

### Statistical analysis

A time-stratified case-crossover design^22^ was applied to examine the association between daily mortality and each PM_2.5_ component. Single-day lags from the current day (lag 0) and 1–3 days prior to death (lag 1, lag 2, and lag 3) were examined separately. In the case-crossover design, within-subject comparisons were made between a case period and control periods. A case period was defined as the date of death. As control periods, we chose the same day of the week in the same month of the same year as the case period. This control selection strategy is expected to adjust for the effects of long-term trends, seasonality, and day of week by design.^23^ We estimated the odds ratios (ORs) and 95% confidence intervals (CIs) of mortality associated with PM_2.5_ mass and each PM component using conditional logistic regression. Based on our previous study,^5^ we used a natural cubic spline function of ambient temperature with 6 degrees of freedom (df) and relative humidity with 3 df for averages from lag 0 to lag 3 (lag 0–3).

First, season-specific estimates were obtained on the effect of PM_2.5_ mass on mortality. The dataset was stratified into summer (June–September), winter (December–March), and transitional seasons (April–May and October–November), in consideration of the temperature distribution. Then, the transitional seasons were further divided into spring (April–May) and autumn (October–November). Second, the effect of each PM component on mortality was estimated using single-pollutant models. Days with missing data for each PM component were excluded from the analysis. In a sensitivity analysis, the df for weather variables were changed. We also adjusted for Ox and NO_2_. Third, we included the multiple PM components simultaneously in multi-pollutant models when the PM components were significantly associated with mortality in the single-pollutant models. We excluded ammonium from multi-pollutant models when sulfate and nitrate were included in the models because ammonium particles generally exists as ammonium sulfate ((NH_4_)_2_SO_4_) and ammonium nitrate ((NH_4_)NO_3_), and ammonium is highly correlated with sulfate and nitrate.^19^ Results were expressed as percent excess risk, which was calculated as (OR − 1) × 100, with a 95% CI of all-cause, cardiovascular, and respiratory mortality per interquartile range (IQR) increase in PM_2.5_ mass and each PM component. This facilitates the comparison of estimated effects among PM components. Analyses were conducted using R (version 3.1.1; R Development Core Team 2014, Vienna, Austria).

## RESULTS

Table 1 displays the total and daily number of deaths for this study. During the study period, the daily number of all-cause deaths varied from 16 to 64 deaths. Table 2 presents the summary statistics of concentrations of PM_2.5_ and its components, as well as weather variables in Nagoya during the study period. There was seasonal variation in the concentration of PM components, with sulfate levels higher in spring and summer (Table 3 and eFigure 2) and nitrate and chloride levels significantly lower in summer because they are usually in the gas phase during warmer seasons. EC and OC levels were highest in autumn. Table 4 shows the correlation coefficients between PM_2.5_ and each chemical component. PM_2.5_ was highly correlated with sulfate (*r* = 0.73), ammonium (*r* = 0.89), potassium (*r* = 0.74), EC (*r* = 0.73), and OC (*r* = 0.78).

**Table 1.** Number of deaths in Nagoya from April 2003 to December 2007

Cause of death	Number of deaths during the study period (1736 days)	Number of deaths for days when PM composition data are available (926 days)	Daily number of deaths during the study period			

			Mean	SD	Minimum	Maximum
All causes except for injuries	61 892	32 969	35.7	(7.4)	16	64
Cardiovascular diseases	20 287	10 850	11.7	(4.0)	1	32
Respiratory diseases	10 437	5591	6.0	(2.7)	0	17

PM, particulate matter; SD, standard deviation.

**Table 2.** Summary statistics of PM_2.5_ and its chemical components in Nagoya from April 2003 to December 2007

Variables	Days of data available	All-year					

		Mean	SD	Minimum	Median	Maximum	IQR
PM_2.5_ (µg/m^3^)	926	23.69	(12.72)	2.55	20.98	77.66	16.24
Sulfate (µg/m^3^)	926	5.57	(4.18)	0.591	4.41	35.23	4.40
Nitrate (µg/m^3^)	926	1.46	(2.05)	0.01 (ND)	0.60	16.00	1.73
Chloride (µg/m^3^)	926	0.28	(0.45)	0.00 (ND)	0.10	4.00	0.34
Ammonium (µg/m^3^)	923	2.61	(1.86)	0.17	2.10	11.67	2.21
Sodium (µg/m^3^)	923	0.15	(0.09)	0.00 (ND)	0.13	0.94	0.11
Potassium (µg/m^3^)	923	0.15	(0.10)	0.00 (ND)	0.12	0.74	0.11
Magnesium (µg/m^3^)	923	0.02	(0.01)	0.00 (ND)	0.02	0.09	0.01
Calcium (µg/m^3^)	923	0.05	(0.05)	0.02 (ND)	0.02	0.59	0.04
Elemental carbon (µg/m^3^)	886	3.55	(2.13)	0.24	3.13	14.55	2.56
Organic carbon (µg/m^3^)	886	4.79	(2.16)	0.88	4.38	21.74	2.40
Ambient temperature (°C)	1736	16.72	(8.31)	−0.33	17.36	32.70	14.81
Relative humidity (%)	1736	65.84	(12.20)	28.00	65.04	96.71	15.88

IQR, interquartile range; ND, not detectable (below the detection limit); PM_2.5_, fine particulate matter; SD, standard deviation.

**Table 3.** Seasonal variation in PM_2.5_ and its chemical components in Nagoya from April 2003 to December 2007

	Spring			Summer			Autumn			Winter		
			
	*n*	Mean (µg/m^3^)	(SD) (µg/m^3^)	*n*	Mean (µg/m^3^)	(SD) (µg/m^3^)	*n*	Mean (µg/m^3^)	(SD) (µg/m^3^)	*n*	Mean (µg/m^3^)	(SD) (µg/m^3^)
PM_2.5_	165	24.56	(11.20)	321	22.56	(11.23)	166	24.86	(14.52)	274	23.79	(13.97)
Sulfate	165	6.39	(3.82)	321	6.75	(5.04)	166	4.47	(3.32)	274	4.35	(3.13)
Nitrate	165	1.83	(2.25)	321	0.56	(1.18)	166	1.37	(1.67)	274	2.33	(2.48)
Chloride	165	0.16	(0.25)	321	0.04	(0.09)	166	0.29	(0.39)	274	0.62	(0.59)
Ammonium	162	2.96	(1.72)	321	2.65	(1.89)	166	2.15	(1.75)	274	2.62	(1.92)
Sodium	162	0.16	(0.09)	321	0.14	(0.10)	166	0.17	(0.09)	274	0.14	(0.08)
Potassium	162	0.16	(0.10)	321	0.12	(0.09)	166	0.17	(0.11)	274	0.16	(0.11)
Magnesium	162	0.02	(0.01)	321	0.02	(0.01)	166	0.02	(0.01)	274	0.02	(0.01)
Calcium	162	0.07	(0.08)	321	0.04	(0.04)	166	0.05	(0.05)	274	0.05	(0.05)
Elemental carbon	148	3.04	(1.84)	309	3.43	(1.68)	155	4.20	(2.62)	274	3.60	(2.35)
Organic carbon	148	4.61	(2.22)	309	4.44	(1.74)	155	5.52	(2.50)	274	4.86	(2.24)

PM_2.5_, fine particulate matter; SD, standard deviation.

**Table 4.** Pearson’s correlation coefficients of PM_2.5_ and its components

	PM_2.5_	Sulfate	Nitrate	Chloride	Ammonium	Sodium	Potassium	Magnesium	Calcium	Elemental carbon	Organic carbon
PM_2.5_	1										
Sulfate	0.73	1									
Nitrate	0.61	0.17	1								
Chloride	0.45	−0.06	0.57	1							
Ammonium	0.89	0.87	0.57	0.27	1						
Sodium	0.26	0.18	0.15	0.20	0.22	1					
Potassium	0.74	0.57	0.44	0.36	0.68	0.37	1				
Magnesium	0.34	0.44	0.07	−0.04	0.37	0.62	0.48	1			
Calcium	0.32	0.24	0.16	0.06	0.25	0.28	0.44	0.49	1		
Elemental carbon	0.73	0.30	0.52	0.56	0.49	0.10	0.43	0.02	0.08	1	
Organic carbon	0.78	0.39	0.43	0.45	0.54	0.19	0.59	0.17	0.19	0.75	1

PM_2.5_, fine particulate matter.

**Figure 1.**
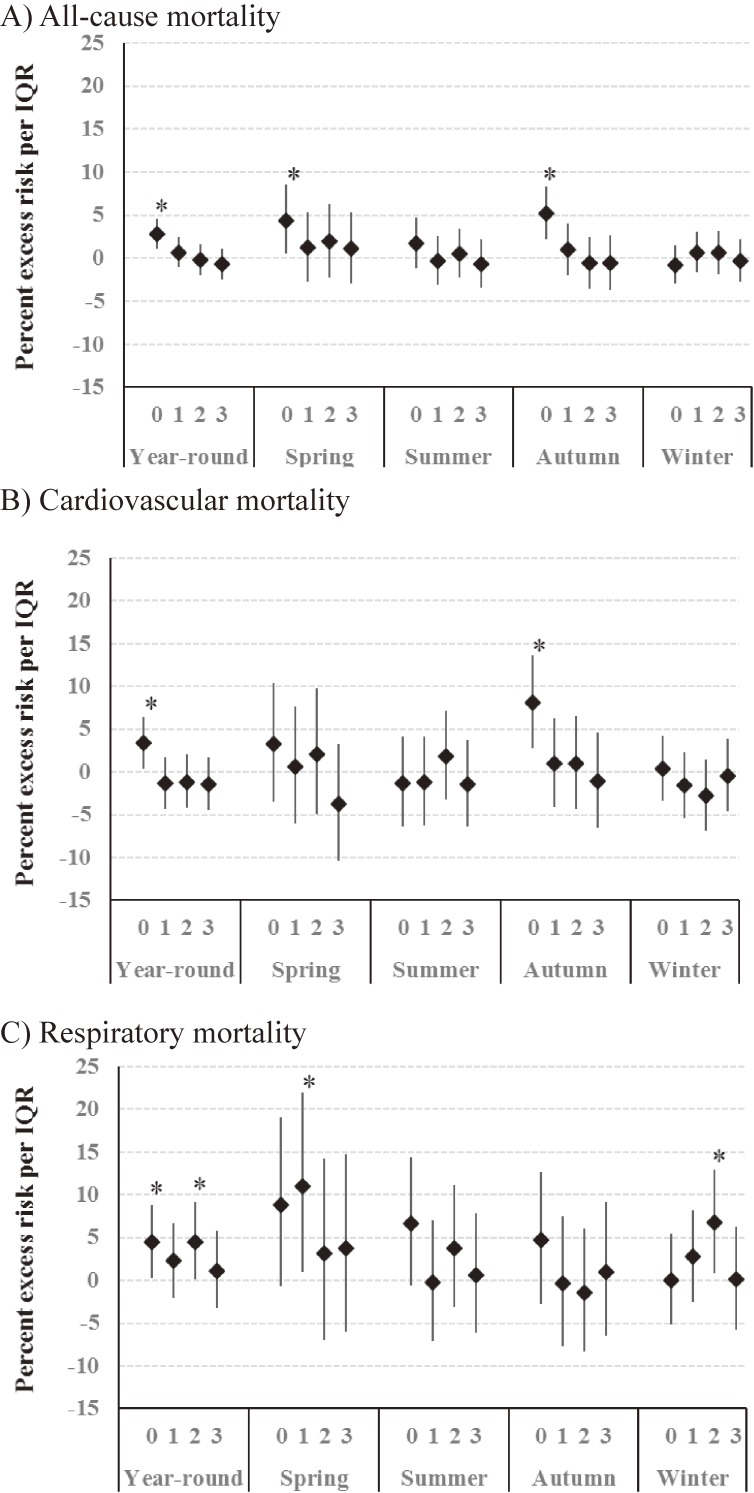
Year-round and season-specific effect estimates of PM_2.5_ mass on (A) all-cause mortality, (B) cardiovascular mortality, and (C) respiratory mortality per IQR increase in PM_2.5_ mass at single-day lags from 0 to 3, adjusted for ambient temperature, and relative humidity. IQR, interquartile range; PM_2.5_, fine particulate matter. *P < 0.05.

Year-round and season-specific estimates of the PM_2.5_ effect are presented in Figure 1. For year-round estimates, an IQR increase in PM_2.5_ mass had associated effects at lag 0 of 2.8% (95% CI, 1.1 to 4.6), 3.4% (95% CI, 0.3 to 6.5), and 4.5% (95% CI, 0.3 to 8.8) on all-cause, cardiovascular, and respiratory mortality, respectively. Association with all-cause mortality was strongest at lag 0 and decreased gradually at later lags. Stronger associations were observed in spring and autumn for all-cause mortality, autumn for cardiovascular mortality, and spring for respiratory mortality.

**Figure 2.**
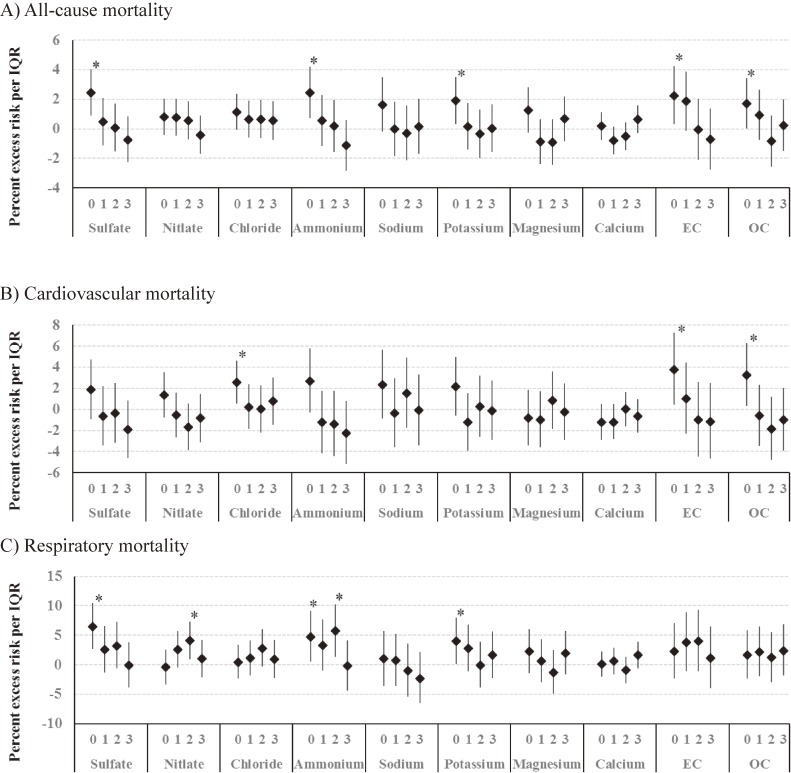
Percent change in all-cause mortality (A), cardiovascular mortality (B), and respiratory mortality (C) per IQR increase in chemical components of PM_2.5_ at single-day lags from 0 to 3, adjusted for ambient temperature, and relative humidity. EC, elemental carbon; IQR, interquartile range; OC, organic carbon; PM_2.5_, fine particulate matter. *P < 0.05.

Figure 2 shows the association between concentrations of the chemical components of PM_2.5_ and mortality in the single-pollutant models. An IQR increase in sulfate was significantly associated with an increase in all-cause mortality (2.4%; 95% CI, 0.9 to 4.0), with a lag pattern similar to that of PM_2.5_ mass. Significant positive associations were also observed for ammonium, potassium, EC, and OC at lag 0. While cardiovascular mortality was significantly associated with chloride, EC, and OC at lag 0, respiratory mortality was significantly associated with sulfate at lag 0, nitrate at lag 2, ammonium at lag 0 and lag 2, and potassium at lag 0.

We changed the df for ambient temperature to 3 and relative humidity to 6, which did not alter results substantially (data not shown). When adjusted for Ox and NO_2_, the positive associations of all-cause mortality with potassium, EC, and OC became non-significant (eFigure 3), whereas the positive associations of cardiovascular mortality with chloride, EC, and OC remained significant. Respiratory mortality was associated only with sulfate. We observed a few inverse associations of all-cause mortality with calcium at lag 1 and OC at lag 2, and cardiovascular mortality with nitrate at lag 2 and OC at lag 2 after adjusting for gaseous pollutants.

**Figure 3.**
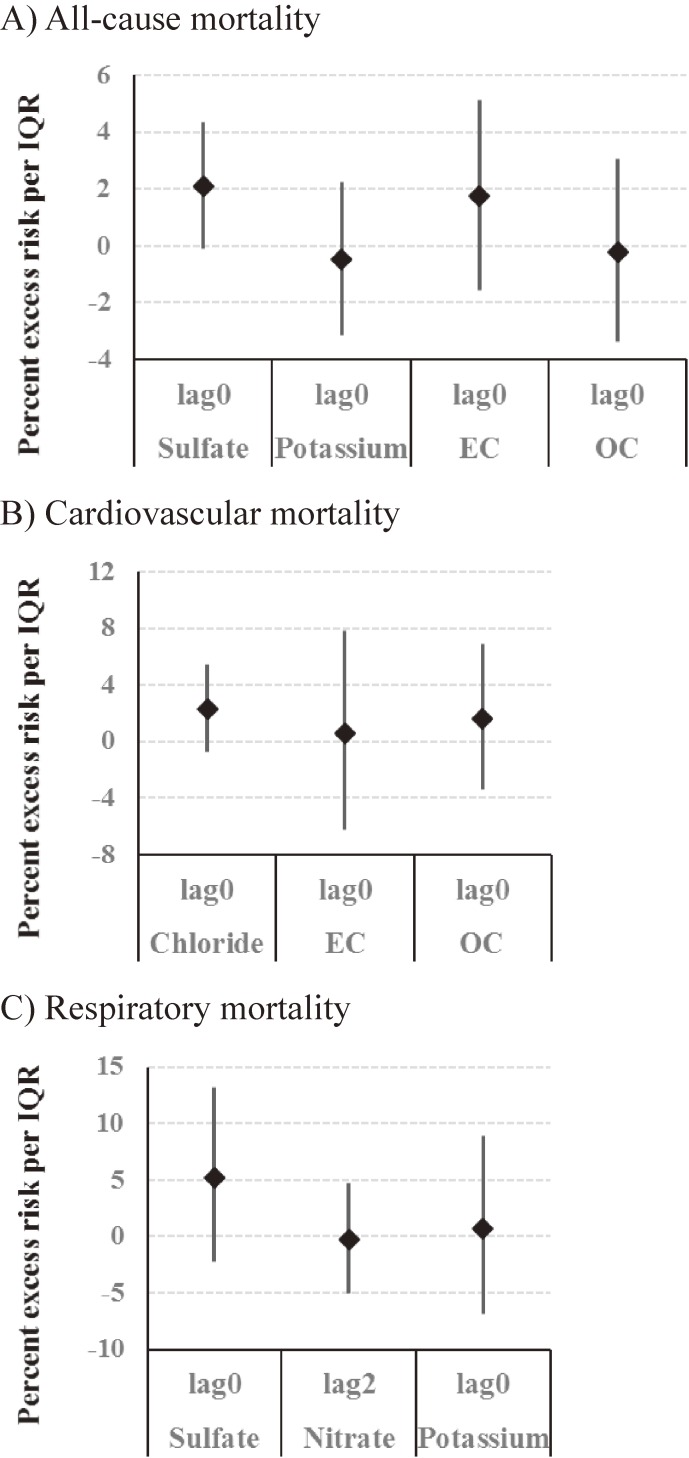
Percent change in all-cause mortality (A), cardiovascular mortality (B), and respiratory mortality (C) per IQR increase in chemical components of PM_2.5_ for the multi-pollutant models. EC, elemental carbon; IQR, interquartile range; OC, organic carbon; PM_2.5_, fine particulate matter.

In the multi-pollutant models, we simultaneously included sulfate, potassium, EC, and OC for all-cause mortality; chloride, EC, and OC for cardiovascular mortality; and sulfate, nitrate, and potassium for respiratory mortality, based on the results of the single-pollutant models. Effect size generally became smaller with wider 95% CIs than in the single-pollutant models. An IQR increase in sulfate was marginally associated with an increase of 2.1% (95% CI, −0.1 to 4.4) in all-cause mortality (Figure 3).

## DISCUSSION

In this study, season-stratified analysis showed a stronger association of PM_2.5_ mass with all-cause mortality in transitional seasons than in summer and winter. We also observed a stronger association of PM_2.5_ mass with cardiovascular mortality in autumn and with respiratory mortality in spring. In an analysis examining the association between mortality and concentrations of each PM component, mortality was significantly associated with sulfate, nitrate, chloride, ammonium, potassium, EC, and OC, which are related to combustion and traffic sources. In the multi-pollutant models, we observed a marginal association between all-cause mortality and sulfate. These findings suggest that some specific PM components are more hazardous to health than others.

Peng et al explored seasonal patterns in the short-term effects of PM on mortality, using 100 cities in the United States.^24^ Their analyses showed that association between PM and mortality varied by region. Stronger associations were observed during spring and summer in northern regions, with no clear seasonal variation in southern regions. They hypothesized that differences in PM composition contributed to the seasonal pattern in association with PM and mortality (ie, there were more toxic particles during warmer seasons in the northern regions). Franklin et al indirectly showed that certain PM components modified the association between PM_2.5_ and mortality in 25 communities in the United States.^12^ They estimated the effect of PM_2.5_ on mortality for each season in each community. Combining seasonal effect estimates and mean seasonal concentration ratios of species to the total PM_2.5_ mass for each community, they used a meta-regression technique to quantify the extent that the association between PM_2.5_ mass and mortality was modified by PM components. Their findings showed that aluminum, sulfate, and nickel modified the association between PM_2.5_ mass and mortality. Our study showed a stronger association between PM_2.5_ mass and mortality in the transitional seasons, when potassium concentrations were higher. Potassium is associated with all-cause and respiratory mortality, and is considered a tracer for biomass burning.^25^ Sulfate and nitrate concentrations also showed clear seasonal variations, although these variations did not necessarily match the seasonal variation in the effect estimates of PM_2.5_ on mortality. It is possible that a certain PM component or combination of several PM components may contribute to the seasonal variation in the health effect of PM_2.5_ mass.

Another possible explanation for the seasonal variation in health effects of PM_2.5_ is the difference in personal exposure by season. More open windows in transitional seasons could contribute to higher air pollutant exposure, which would lead to a larger effect.^8^

Our analysis suggests that sulfate is associated with all-cause and respiratory mortality. Sulfate secondary particles are produced by the oxidation of sulfur dioxide and nitrogen oxides in the atmosphere,^26^ and the concentration of sulfate in Japan is influenced by transboundary pollution outflow from the Asian continent.^27^ The lag pattern of association between sulfate and mortality was similar to the pattern between PM_2.5_ mass and mortality. It is possible that the amount of inhaled particles, but not a specific component, influenced the size of the effect because sulfate composes a large fraction of and is highly correlated with total PM_2.5_ mass concentration. To take mass concentration into account, we adjusted for PM_2.5_ mass concentration (eFigure 4). Although the positive association between sulfate and all-cause mortality became non-significant, the association between sulfate and respiratory mortality remained significant. This result suggests that sulfate is responsible for PM toxicity.

While several epidemiological studies have shown a significant association between sulfate and mortality, toxicological studies have not fully provided evidence indicating plausible biological mechanisms for this association.^26^ There are a few possible explanations for the association between sulfate and mortality. First, the acidity of sulfate particles may mediate adverse health effects. Dreher et al suggested that particle acidity may contribute to adverse health effects by altering the pulmonary toxicity of other components or physical properties instead of through its own direct toxicity.^28^ Second, sulfate may catalyze metals to become bioavailable, which consequently causes oxidative stress.^29^ Third, sulfate may be a proxy of unmeasured PM components. Previous epidemiological studies gave heterogeneous results on the association between particle components and mortality. Some studies found positive associations for sulfate^1^^,^^15^^,^^20^^,^^30^^,^^31^ and nitrate,^14^^,^^15^ while at least one did not.^13^ Further evidence is required to confirm plausible mechanisms.

Nitrate was associated with respiratory mortality. Based on the concentration levels showing both diurnal and seasonal variations, nitrate is likely produced by photochemical reactions.^21^ There are a few epidemiological studies on the health effects of nitrate. Cao et al found that nitrate was associated with total and cardiovascular mortality in Xi’an, a heavily polluted city in China.^20^ Ostro et al also observed a strong association of nitrate with cardiovascular mortality in California.^14^ In a study of 72 urban communities in the United States, however, nitrate was not significantly associated with mortality.^13^ No single PM component, but rather a combination of multiple components, appear responsible for the health effects of PM.

EC and OC were significantly associated with all-cause and cardiovascular mortality. Our findings are consistent with previous studies.^14^^,^^16^^,^^19^^,^^20^ Ostro et al found that EC and OC were associated with cardiovascular mortality in California.^14^ Zhou et al examined the association of mortality and PM components in Detroit and Seattle and observed associations of cardiovascular mortality with EC and OC only in the cold season in Seattle.^16^ In Asian countries, Heo et al^19^ and Cao et al^20^ reported that OC was associated with cardiovascular mortality, whereas Son et al^18^ observed no significant associations of cardiovascular mortality with EC or OC. The major source of EC and OC is biomass and fossil fuel combustion. It is possible that EC and OC reflect the effects of other correlated components. Previous studies found that combustion-related metals, such as nickel and copper, were associated with mortality.^14^^,^^20^

Although ammonium was found to be associated with mortality, this may reflect the association of sulfate and nitrate with mortality. In the atmosphere, ammonium salts, such as (NH_4_)_2_SO_4_ and (NH_4_)NO_3_, are formed in the presence of ammonia gas due to acid gas neutralization.^19^ Therefore, the concentration of ammonium is highly correlated with the sum of sulfate and nitrate. Most sulfate and nitrate particles are secondary particles produced by atmospheric photochemical reactions of gaseous pollutants.^20^ These gaseous pollutants are mainly emitted due to fuel combustion. Therefore, the association of these components and respiratory mortality indicate that combustion-related particles have a greater toxic effect on health than particles from other sources.

After adjustment for co-pollutants, the lag pattern varied widely. Like other single-city studies,^19^^,^^20^ we observed significant inverse associations between mortality and some of the PM components. This may be partially explained by the influence of multicollinearity, given that several PM components are highly correlated with PM_2.5_ mass and gaseous pollutants.

This study has several limitations worth noting. First, the chemical components of PM_2.5_ were only monitored from Sunday to Thursday. We evaluated single-day lags separately. Hence, the effect estimate was calculated using different days for each lag. This could have affected our effect estimates because the distribution of PM composition differs between weekdays and the weekends.^32^ The concentrations of PM_2.5_ mass and several PM components were lower on Sunday than on weekdays in our study (eTable 1). Further studies including the data both of weekdays and weekends are needed. Second, we used PM components sampled from 9:30 a.m. to 9:00 a.m. of the next day as a proxy of concentration at lag 0. We assumed that PM_2.5_ concentrations sampled from 9 a.m. to 9 a.m. of the next day would reflect concentrations from 0 to 23 hours on that day because these concentrations were highly correlated. However, this assumption may not be applicable to some PM components emitted from local sources. The effect estimates at lag 0 for PM components may have been influenced by temporal exposure misclassification and may be less reliable than those at lag 1. Third, we used the data from a single monitoring site as a proxy of personal exposure to PM_2.5_ and its components in Nagoya City. Although the measured PM_2.5_ concentrations at the study site were considered to represent those in the southern area of Nagoya City, random measurement errors might have been introduced, possibly resulting in bias toward the null in effect estimates. Fourth, as this was a single-city study, our findings may not be generalizable to cities with a different mixture of air pollutants. Thus, further studies should be conducted in other areas of Japan.

In conclusion, we found that sulfate, nitrate, chloride, ammonium, potassium, EC, and OC are associated with mortality in Nagoya, Japan. Our findings suggest that some specific PM components have more hazardous effects than others and contribute to seasonal variation in the health effects of PM_2.5_.

## ONLINE ONLY MATERIALS

eFigure 1. Scatter plot of 24-hour mean concentrations of PM_2.5_ from 12 a.m. of the day until 12 a.m. of the next day and those from 9 a.m. of the day until 9 a.m. of the next day (*n* = 346) calculated from the hourly sampling obtained using a Tapered Element Oscillating Microbalance in 2003 at the site, which is 6.5 km from the study site in Nagoya.

eFigure 2. Monthly variation in PM_2.5_ mass and its components in Nagoya from April 2003 to December 2007. Box plots represent the median (horizontal line) and the 25th and 75th percentiles (edges of box).

eFigure 3. Percent change in all-cause mortality (A), cardiovascular mortality (B), and respiratory mortality (C) per IQR increase in chemical components of PM_2.5_ at single-day lags from 0 to 3, adjusted for ambient temperature, relative humidity, Ox, and NO_2_.

eFigure 4. Percent change in all-cause mortality (A), cardiovascular mortality (B), and respiratory mortality (C) per IQR increase in chemical components of PM_2.5_ at single-day lags from 0 to 3, adjusted for ambient temperature, relative humidity, Ox, NO_2_, and PM_2.5_ mass.

eTable 1. Average and standard deviation of PM_2.5_ mass and its components for each day of the week in Nagoya from April 2003 to December 2007.

Abstract in Japanese.
