# Supplementary material for: Associations Between Fine Particulate Matter Components and Daily Mortality in Nagoya, Japan
Source: J Epidemiol. 2016 May 5;26(5):249–57. doi: 10.2188/jea.JE20150039 (PMC4848323; doi:10.2188/jea.JE20150039)
Supplement: eFigure 1. [file je-26-249-s001.pdf]

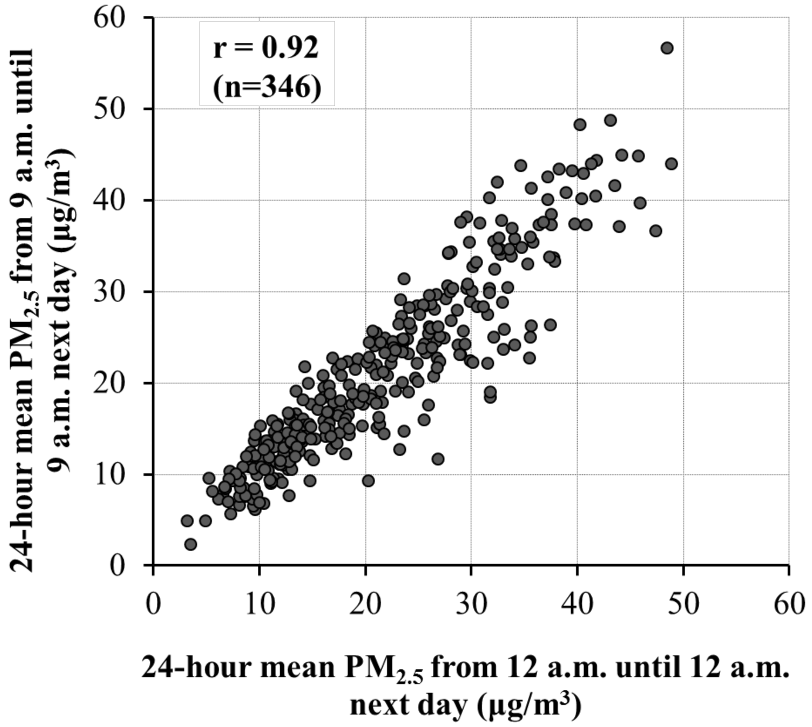


**eFigure 1.** Scatter plot of 24-hour mean concentrations of $PM_{2.5}$ from 12 a.m. of the day until 12 a.m. of the next day and those from 9 a.m. of the day until 9 a.m. of the next day (n=346) calculated from the hourly sampling obtained using a Tapered Element Oscillating Microbalance in 2003 at the site, which is 6.5 km from the study site in Nagoya.
