# Supplementary material for: Associations Between Fine Particulate Matter Components and Daily Mortality in Nagoya, Japan
Source: J Epidemiol. 2016 May 5;26(5):249–57. doi: 10.2188/jea.JE20150039 (PMC4848323; doi:10.2188/jea.JE20150039)
Supplement: eFigure 2. [file je-26-249-s002.pdf]

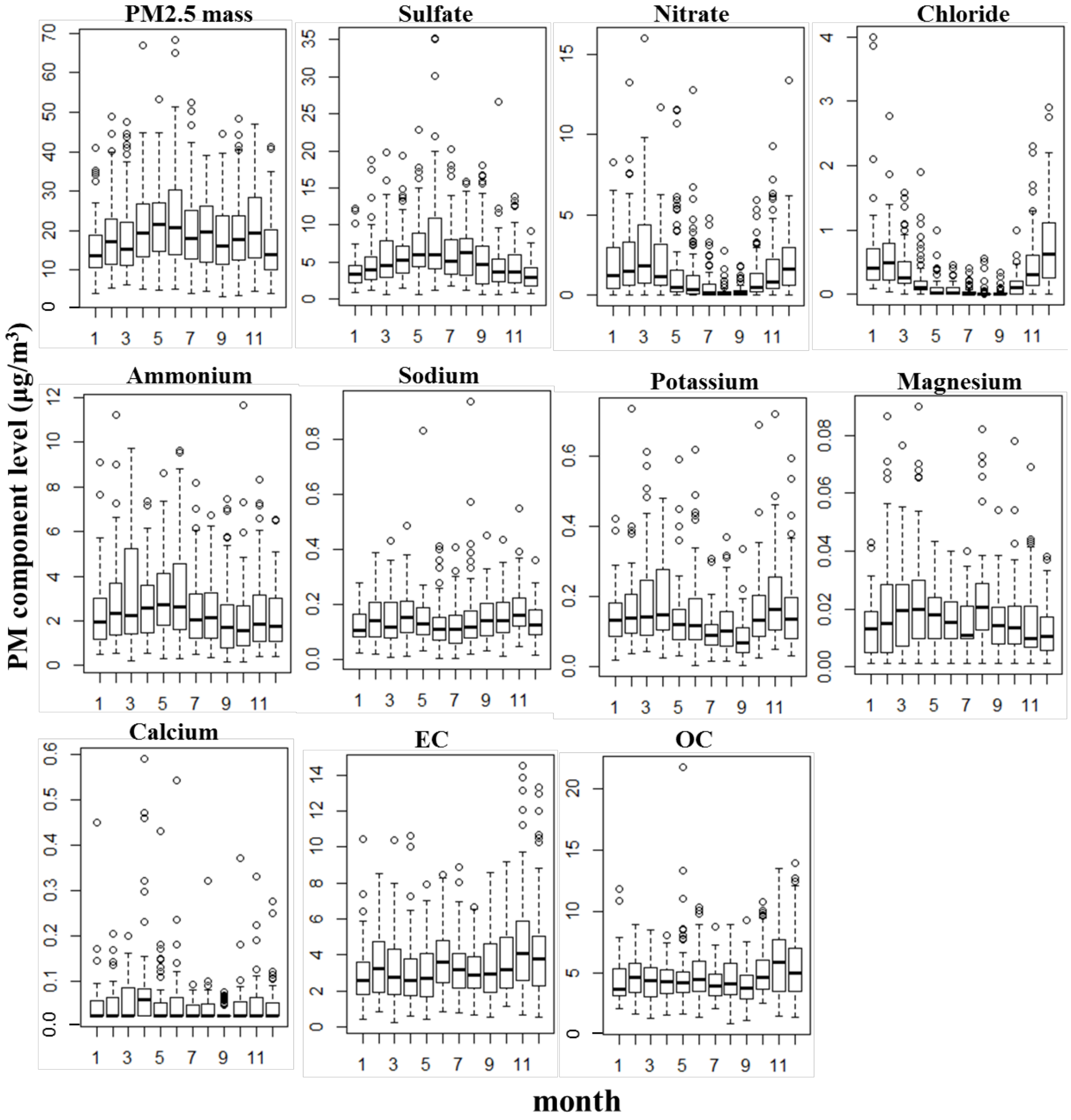

eFigure 2. Monthly variation in $PM_{2.5}$ mass and its components in Nagoya from April 2003 to December 2007. Box plots represent the median (horizontal line) and the 25th and 75th percentiles (edges of box).
