# Supplementary material for: Associations Between Fine Particulate Matter Components and Daily Mortality in Nagoya, Japan
Source: J Epidemiol. 2016 May 5;26(5):249–57. doi: 10.2188/jea.JE20150039 (PMC4848323; doi:10.2188/jea.JE20150039)
Supplement: eFigure 3. [file je-26-249-s003.pdf]

A) All-cause mortality

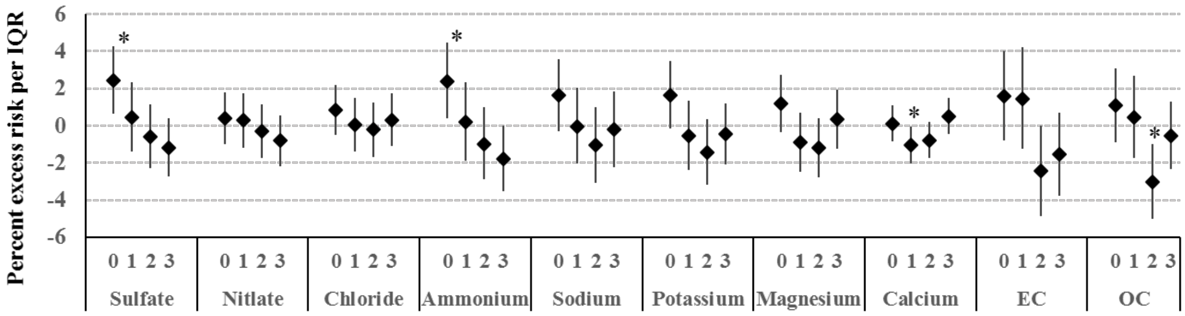

B) Cardiovascular mortality

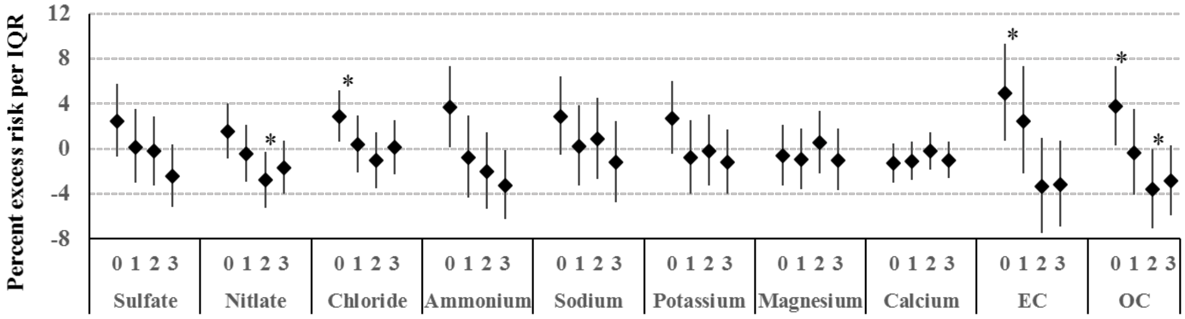

C) Respiratory mortality

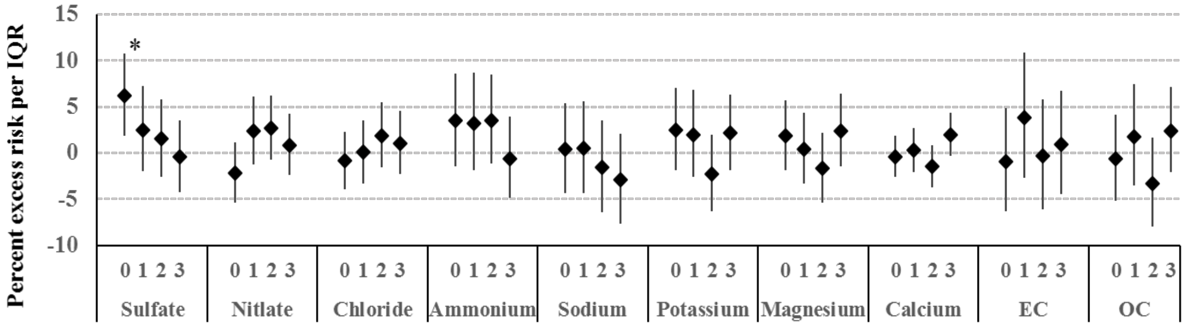

eFigure 3. Percent change in all-cause mortality (A), cardiovascular mortality (B), and respiratory mortality (C) per IQR increase in chemical components of $PM_{2.5}$ at single-day lags from 0 to 3, adjusted for ambient temperature, relative humidity, Ox, and NO_2.

EC, elemental carbon; IQR, interquartile range; OC, organic carbon; $PM_{2.5}$, fine particulate matter.

* P<0.05
