# Supplementary material for: Associations Between Fine Particulate Matter Components and Daily Mortality in Nagoya, Japan
Source: J Epidemiol. 2016 May 5;26(5):249–57. doi: 10.2188/jea.JE20150039 (PMC4848323; doi:10.2188/jea.JE20150039)
Supplement: eTable 1. [file je-26-249-s005.pdf]

eTable 1. Average and standard deviation of $PM_{2.5}$ mass and its components for each day of the week in Nagoya from April 2003 to December 2007

Variables	Mean (SD)					
	Sunday (n=165)	Monday (n=181)	Tuesday (n=195)	Wednesday (n=184)	Thursday (n=182)	Friday (n=18)
$PM_{2.5}$ (μg/m^3)	19.89 (11.06)	23.83 (12.32)	23.76 (13.06)	24.72 (12.25)	24.90 (13.44)	33.15 (16.36)
Sulfate (μg/m^3)	4.93 (3.57)	5.24 (3.33)	5.39 (4.17)	5.95 (4.52)	6.10 (4.94)	7.18 (4.38)
Nitrate (μg/m^3)	1.18 (1.68)	1.47 (2.11)	1.44 (1.93)	1.57 (2.21)	1.48 (2.11)	2.66 (3.17)
Chloride (μg/m^3)	0.17 (0.25)	0.30 (0.47)	0.32 (0.54)	0.29 (0.45)	0.28 (0.44)	0.45 (0.51)
Ammonium (μg/m^3)	2.27 (1.60)	2.50 (1.70)	2.55 (1.85)	2.76 (1.88)	2.82 (2.07)	3.66 (2.58)
Sodium (μg/m^3)	0.13 (0.08)	0.15 (0.09)	0.14 (0.09)	0.15 (0.10)	0.15 (0.10)	0.16 (0.06)
Potassium (μg/m^3)	0.13 (0.09)	0.15 (0.10)	0.14 (0.11)	0.15 (0.11)	0.16 (0.10)	0.21 (0.10)
Magnesium (μg/m^3)	0.02 (0.01)	0.02 (0.01)	0.02 (0.01)	0.02 (0.01)	0.02 (0.02)	0.02 (0.02)
Calcium (μg/m^3)	0.04 (0.07)	0.05 (0.06)	0.05 (0.05)	0.05 (0.04)	0.05 (0.05)	0.07 (0.05)
Elemental carbon (μg/m^3)	2.25 (1.30)	3.71 (2.09)	3.74 (2.11)	3.91 (2.12)	3.84 (2.24)	5.09 (3.24)
Organic carbon (μg/m^3)	4.22 (1.95)	4.87 (2.36)	4.73 (2.03)	4.89 (1.97)	4.97 (2.21)	6.54 (2.95)

$PM_{2.5}$, fine particulate matter; SD, standard deviation.
