# Supplementary material for: Associations Between Fine Particulate Matter Components and Daily Mortality in Nagoya, Japan
Source: J Epidemiol. 2016 May 5;26(5):249–57. doi: 10.2188/jea.JE20150039 (PMC4848323; doi:10.2188/jea.JE20150039)
Supplement: Abstract in Japanese. [file je-26-249-s006.pdf]

名古屋における微小粒子状物質成分と日死亡との関連

上田佳代[1], 山神真紀子[2], 池盛文数[2], 久恒邦弘[2], 新田裕史[3]
[1]京都大学大学院工学研究科, [2]名古屋市環境科学調査センター、[3]国立環境研究所

背景：
微小粒子状物質(PM2.5)の死亡に対する影響推定値には、季節変動や地域差がみられ、この変動には PM2.5 の化学成分の違いが関係している可能性がある。本研究では、名古屋市において PM2.5 の成分濃度と死亡との関連について検討した。
方法：
名古屋市の 2003 年 4 月から 2007 年 12 月までにおける 65 歳以上の日死亡数と PM2.5 重量濃度及び成分濃度のデータを結合した。時間層別化ケースクロスオーバーデザインを用い、日々の死亡と PM2.5 重量濃度及びそれぞれの成分（塩化物イオン、硝酸イオン、硫酸イオン、ナトリウムイオン、カリウムイオン、カルシウムイオン、マグネシウムイオン、アンモニウムイオン、元素状炭素、有機炭素）との関連について検討した。
結果：
PM2.5 重量濃度と死亡との関連は、春・秋により大きかった。PM2.5 成分濃度については、単汚染物質モデルで、硫酸イオン、硝酸イオン、塩化物イオン、アンモニウムイオン、カリウムイオン、元元素状炭素、有機炭素が死亡と有意な関連がみられた。多汚染物質モデルでは、硫酸イオンの四分位範囲濃度上昇あたり、2.1%(95%信頼区間、-0.1, 4.4%)の全死亡増加と関連しており、有意に近い関連であった。
結論：
これらの結果から、ある粒子状物質の成分は他の成分より有害な影響が大きく、粒子状物質の健康影響の四季変動に関わっていることを示唆した。

キーワード：粒子状物質、化学成分、死亡、大気汚染
